# Is xenodontine snake reproduction shaped by ancestry, more than by ecology?

**DOI:** 10.1002/ece3.2557

**Published:** 2016-12-20

**Authors:** Gisela P. Bellini, Vanesa Arzamendia, Alejandro R. Giraudo

**Affiliations:** ^1^ Instituto Nacional de Limnología (CONICET‐UNL)Santa FeArgentina; ^2^ Facultad de Humanidades y CienciasUniversidad Nacional del LitoralSanta FeArgentina

**Keywords:** canonical phylogenetic ordination, ecological factors, evolutionary ecology, life‐history, phylogenetic history

## Abstract

One of the current challenges of evolutionary ecology is to understand the effects of phylogenetic history (PH) and/or ecological factors (EF) on the life‐history traits of the species. Here, the effects of environment and phylogeny are tested for the first time on the reproductive biology of South American xenodontine snakes. We studied 60% of the tribes of this endemic and most representative clade in a temperate region of South America. A comparative method (canonical phylogenetic ordination—CPO) was used to find the relative contributions of EF and PH upon life‐history aspects of snakes, comparing the reproductive mode, mean fecundity, reproductive potential, and frequency of nearly 1,000 specimens. CPO analysis showed that PH or ancestry explained most of the variation in reproduction, whereas EF explained little of this variation. The reproductive traits under study are suggested to have a strong phylogenetic signal in this clade, the ancestry playing a big role in reproduction. The EF also influenced the reproduction of South American xenodontines, although to a lesser extent. Our finding provides new evidence of how the evolutionary history is embodied in the traits of living species.

## 1 Introduction

Ecology and evolutionary biology have remained separate for many years, but now it is recognized that both disciplines are almost inseparable. For this purpose, comparisons among species are one of the most widely used methodologies in all areas of evolutionary biology (e.g., Bellini, Giraudo, Arzamendia, & Etchepare, 2015; Colston, Costa, & Vitt, 2010; Morales & Giannini, 2010; Tulli, Cruz, Herrel, Vanhooydonck, & Abdala, 2009; Vera Candioti & Altig, 2010). The analysis of interspecific variation in life‐history traits provides a basis for understanding evolutionary patterns and remains a major tool for evolutionary biologists (Pizzatto, Almeida‐Santos, & Shine, 2007a; Zuffi et al., 2009). In particular, interspecific comparisons allow us to understand how ancestral heritage (phylogenetic inertia) and natural selection (adaptation) have molded the features we observe in the present species. Such analyses explicitly recognize that species share many characteristics as a consequence of their common ancestry (Freckleton, 2000). In other words, ecological data for species are compared in a phylogenetic framework to investigate whether a relationship exists among ecological and phylogenetic similarities (Losos, 2008). Some of the strongest suggestive evidence of adaptation comes from empirical patterns of covariation between morphology, reproductive biology, and general ecology (e.g., habitat use). Such patterns can allow strong inferences about the selective forces that have shaped life‐history diversity (Pizzatto, Almeida‐Santos, & Marques, 2007b).

Westoby, Leishman, and Lord (1995) initiated a controversy about what they called “phylogenetic correction” which is the control for phylogeny in comparative analyses. The comparative methods partition explained variation of ecological data in such a way that they allocate the maximum possible variation in a trait to phylogeny considering only the residual as potentially attributable to ecology (Westoby et al., 1995). The technique of variation partitioning is used when two or more complementary sets of hypotheses can be invoked to explain the variation of a response variable (Legendre, 2007). Indeed, the phylogenetic portion of the total variance of the variable of interest may contain components related to ecology, so a portion of that variance would no longer be exclusive to phylogeny, but it would be a shared variance between this and the ecology. This method controls for the phylogenetic component in the variables when estimating the influence of present‐day ecological factors. This is justified by the principle of parsimony, because related species do share phylogenetic history, and our interest is to quantify how other factors account for the distribution of characters (Desdevises, Legendre, Azouzi, & Morand, 2003).

Most aspects of ectotherms (morphology, behavior, physiology, and reproduction) are strongly influenced by environmental factors (Zuffi et al., 2009). This makes snakes appropriate organisms for comparative studies, in which phylogenetic history (PH) versus ecological factors (EF) are contrasted. In this work, we tested the effects of environment and phylogeny on the reproductive biology of South American xenodontine snakes. Reproduction is perhaps the single most important biological function as it is the means by which organisms transmit genes to the next generation (Van Dyke, Brandley, & Thompson, 2014). As reproduction is a major component of an organism's life history, elucidating reproductive characters is essential for understanding the animal life cycle (Almeida‐Santos et al., 2014; Pizzatto, Jordao, & Marques, 2008a; Shine, 2003). Reproduction in reptiles is influenced by ecological, environmental, phylogenetic, and geographical factors (Cadle & Greene, 1993; Di‐Bernardo, 1998; Giraudo, Arzamendia, & López, 2007; Gregory & Larsen, 1993). The strong causal link between life‐history traits and individual reproductive success has encouraged many researchers to look for an adaptive basis to variation in life‐history traits. That search has revealed immense diversity among taxa, with some traits exhibiting strong phylogenetic conservatism among major lineages, whereas other traits display remarkable convergence and parallelism (Shine, 2005).

Reproductive biology of snakes includes different factors, such as reproductive mode, reproductive cycles, fecundity, age and size at maturity, sexual dimorphism, mating systems, and reproductive behavior (Almeida‐Santos et al., 2014). Generally, variation in those reproductive characteristics in squamates is attributed to different EF, such as climatic conditions and/or food availability (see Barros, Rojas, & Almeida‐Santos, 2014; Gregory, 2009; Shine, 2003). On the other hand, the absence of variability in these traits among populations of reptiles that live under different climatic conditions is commonly attributable to the influence of PH, even without performing analyses (see Barros, Sueiro, & Almeida‐Santos, 2012; Shine, 2005). According to Shine (2003), female snakes coordinate their reproductive decisions with temporal fluctuations in energy availability. In these organisms, the costs of reproduction impose strong selection pressure, forcing them to adjust their reproductive strategies to local conditions. Abiotic, ecological, and geographical factors condition sexual maturity, fecundity, and sexual dimorphism, generating divergence in reproductive tactics among and within species (Shine, 2003; Vitt & Vangilder, 1983). For example, particular adjustments were observed in the reproductive types and phenology when species of aquatic and terrestrial snakes were compared in an extensive study in the Middle Paraná River (Giraudo et al., 2007). This research showed that the use of habitat could influence the reproductive characteristics of snakes. On the other hand, characters related to reproduction in Neotropical snakes seem to be relatively conservative in some phylogenetic lineages, although in other groups even closely related species may differ widely in their reproductive ecology (Pizzatto et al., 2008b).

Even the reproductive mode is generally phylogenetically constrained in snakes, which reproduce either by laying eggs (oviparity) or by giving birth to live young (viviparity) (Feldman et al., 2015). In some studies, the influence of both EF and PH was recognized in reptile reproduction (Cadle & Greene, 1993; Di‐Bernardo, 1998; Gregory & Larsen, 1993). However, despite this recognition, it was not actually measured in snakes. Some authors have recently begun to incorporate some phylogenetic explanations for certain reproductive characteristics, although there are still different views regarding this topic and much remains to be investigated. Pizzatto et al. (2008a), for example, stated that clutch size, duration of vitellogenesis, and egg‐carrying period are likely to be conditioned by phylogenetic factors, but they do not report any analyses about this topic. Something similar happens with the studies of Barros et al. (2012, 2014) in which they proposed phylogenetic inertia for some reproductive patterns or strategies without any specific analyses. They even argue, without analytical support, that the timing of female reproductive events in snakes may be conservative in a lineage, despite being influenced by factors such as climate conditions and food availability.

Most studies of snakes in South America were focused primarily on the natural history of the species, and research on the effects of ecology and evolutionary history is still scarce (França, Mesquita, Nogueira, & Araújo, 2008). Moreover, most of these studies only analyzed traits such as diet and morphology; only one contribution has been made considering ecological and phylogenetic aspects of South American squamate reproduction, but it was conducted on subtropical lizards (Mesquita & Colli, 2010).

Temperate environments of South America are particularly useful for the investigation of patterns of reproductive cycles and the effects of environment on the reproductive biology of snakes (Mesquita, Mattos, Sá‐Polidoro, & Cechin, 2013). Our key question is to what extent ecological factors or phylogenetic relationships influence or interact in the major reproductive attributes of snakes.

We focus in Xenodontinae as a model because is one of the largest subfamilies of snakes, all restricted to the New World and characterized by a great morphological and ecological diversity (Cadle, 1984; Cadle & Greene, 1993; Grazziotin et al., 2012; Vidal, Kindl, Wong, & Hedges, 2000). Even more, it appears that each xenodontine lineage (North, Central, and South American xenodontines) is able to invade many ecological niches (Vidal et al., 2000). Although the chasm between the ecological and phylogenetic contributions to species characters seems to be extensive, resolving this controversy will undoubtedly play a huge role in the development of evolutionary ecology. With this purpose in mind, we have continued the study of temperate South American snake communities already started in previous studies. To this end, we tested the phylogenetic and ecological influence on the reproduction in snakes, using comparative methods that combined life‐history data with current phylogenetic hypotheses. In this opportunity, we focus on the South American xenodontines, or Xenodontines sensu stricto, which comprise the Dipsadidae, a Neotropical endemic family proposed by Zaher et al. (2009). The analysis included 60% of the tribes of this endemic and most representative clade in South America.

## 2 Materials and Methods

### 2.1 Study area

The field study was carried out in a temperate area of South America between 24°30′S and 35°30′S latitude, and 65°′W and 53°W longitude, a region in the Chacoan dominion (sensu Morrone, 2014). This area is characterized by a mosaic of vegetation ranging from savannas and grasslands to temperate deciduous forests, with a wide variety of wetlands. The geomorphology and landscape of this area have been strongly influenced by the three large South American rivers of the Plata Basin—the Paraná, Uruguay, and Paraguay Rivers—that converge to form the La Plata River. Cabrera (1994) and Morrone (2014) described phytogeographical and zoogeographical aspects of the region. The climate is seasonal, with a hot and rainy spring (mean temperature: 25°C) and summer (mean temperature: 27.5°C) and a dry autumn (mean temperature: 15°C) and winter (mean temperature: 10°C). Precipitation decreases from northeast to southeast, and annual precipitation ranges from 800 to 1,800 mm (Iglesias de Cuello, 1982; Paoli, Iriondo, & García, 2000).

### 2.2 Reproductive data collection

Reproductive data were obtained by analyzing 918 adult females belonging to 17 species of snakes, which belonged to nine genera and six tribes of the subfamily Xenodontinae. The number of individuals from each species is shown in Table 1. The study area was sampled from January 1991 to April 2014, mainly by means of road sampling and time‐constrained searches in different habitats. Recently, road‐killed snakes that were in good condition were preserved for collecting reproductive data. All collected specimens are housed in the collection of the Instituto Nacional de Limnología (INALI, Santa Fe, Argentina). The material was supplemented with data from specimens deposited in the following scientific collections: Museo Argentino de Ciencias Naturales “Bernadino Rivadavia” (MACN, Buenos Aires), Colección del Museo de La Plata (MLP, Buenos Aires), Museo Antonio Serrano (MAS, Entre Ríos), Universidad Nacional del Nordeste (UNNE, Corrientes), and Museo Provincial de Ciencias Naturales “Florentino Ameghino” (MFA, Santa Fe).

**Table 1:** Reproductive and ecological attributes of 17 species in a temperate South American snake community

Tribe	Species	*N*	R M	R F	R P	M F	SU	HU
Philodryadini	*Philodryas aestiva*	21	O	0.8	M	10	T	S
	*Philodryas olfersii*	22	O	0.5	L	7	A	FO
	*Philodryas patagoniensis*	118	O	0.9	H	12	T	S
Tachimenini	*Thamnodynastes chaquensis*	48	V	0.8	M	11	T	W
	*Thamnodynastes hypoconia*	67	V	0.5	L	8	AQ	W
	*Thamnodynastes strigatus*	20	V	0.5	M	11	AQ	W
Hydropsini	*Helicops infrataeniatus*	65	V	0.5	M	17	AQ	W
	*Helicops leopardinus*	95	V	0.5	M	15	AQ	W
Hydrodynastini	*Hydrodynastes gigas*	77	O	0.7	H	23	AQ	W
Pseudoboini	*Paraphimophis rustica*	20	O	0.6	M	9	T	S
	*Boiruna maculata*	18	O	0.5	L	8	T	S
Xenodontini	*Erythrolamprus jaegeri*	21	O	0.6	L	6	T	G
	*Erythrolamprus poecilogyrus*	93	O	0.6	M	8	T	G
	*Erythrolamprus semiaureus*	90	O	0.9	H	14	AQ	W
	*Lygophis anomalus*	62	O	0.5	L	7	T	S
	*Xenodon dorbingyi*	32	O	0.7	M	11	F	S
	*Xenodon merremii*	49	O	0.8	H	16	T	G

References: A, arboreal; AQ, aquatic; F, fossorial; Fo, forest; G, generalist; H, high; HU, habitat use; L, low; M, medium; MF, mean fecundity; *N*, number of individuals; O, oviparous; RF, reproductive frequency; RM, reproductive mode; RP reproductive potential; S, savanna; SU, substrate use; T, terrestrial; V, viviparous; W, wetland.

Snakes were sexed by direct examination of gonads. Snakes with vitellogenic follicles, oviductal eggs, or folded oviducts were considered to be mature and were used for the analysis (see Leite, Nunes, Kaefer, & Cechin, 2009; Pizzatto et al., 2007a, 2008a). The diameter of the largest ovarian follicle or oviductal egg (length and width in mm) was recorded using a digital caliper. The snout–vent length (SVL) was measured with a flexible ruler (in mm). We defined the reproductive season as the period from secondary vitellogenesis to oviposition or the bearing of young (Leite et al., 2009). Reproductive frequency (RF) was estimated by the percentage of reproductive females in the sample (Pizzatto, 2005). Fifty percentage or less of mature females with no vitellogenic follicles or eggs in the reproductive season was an evidence of biannual or multiannual reproductive cycle (Bellini, Arzamendia, & Giraudo, 2013). By contrast, an annual frequency is assumed when more than 50% of the population of mature females are reproductively active in the breeding season. Oviductal eggs and embryos were counted to estimate the mean fecundity (Almeida‐Santos et al., 2014; Pizzatto, 2005). The reproductive potential (RP), which shows the number of potential neonates of one species per female per year, was estimated as mean fecundity × reproductive frequency (Trauth, 1978). The RP was classified into the following categories: low, when the value was less than five; medium, when the value was more than five and less than ten; and high, when the value was more than ten.

### 2.3 Statistical analysis

Following the methodology proposed by Morales and Giannini (2010), we first carried out a redundancy analysis (RDA) to test whether the use of the substrate and habitat (ecological variables) was associated with reproductive variation. RDA is an ordination technique deriving from PCA, with a linear constraint represented by explanatory variables of an external matrix (ter Braak, 1995). In our study, the main matrix is represented by reproductive variables: mean fecundity, reproductive mode (oviparous, viviparous), reproductive frequency (annual, biennial), and reproductive potential (low, medium, high), hereafter termed the “reproduction matrix.” The external matrix is represented by the assignment of the 17 species to each of the categories from the ecological classifications, hereafter termed the “ecological matrix.” The ecological matrix was made with published information from field studies in the study area (Arzamendia & Giraudo, 2009; Bellini, Giraudo, & Arzamendia, 2014; Bellini et al., 2013; Giraudo, 2001; Giraudo, Arzamendia, Bellini, Bessa, & Costanzo, 2014; Giraudo et al., 2007). The ecological variables were associated with the use of substrate (aquatic, terrestrial, arboreal, fossorial) and use of habitat (forest, wetland, savanna, generalist). Significance was evaluated using 9999 unrestricted Monte Carlo permutations for individual ecological categories, using forward stepwise addition. In all cases, the alpha level of significance was set at 0.05.

**Figure 1:**
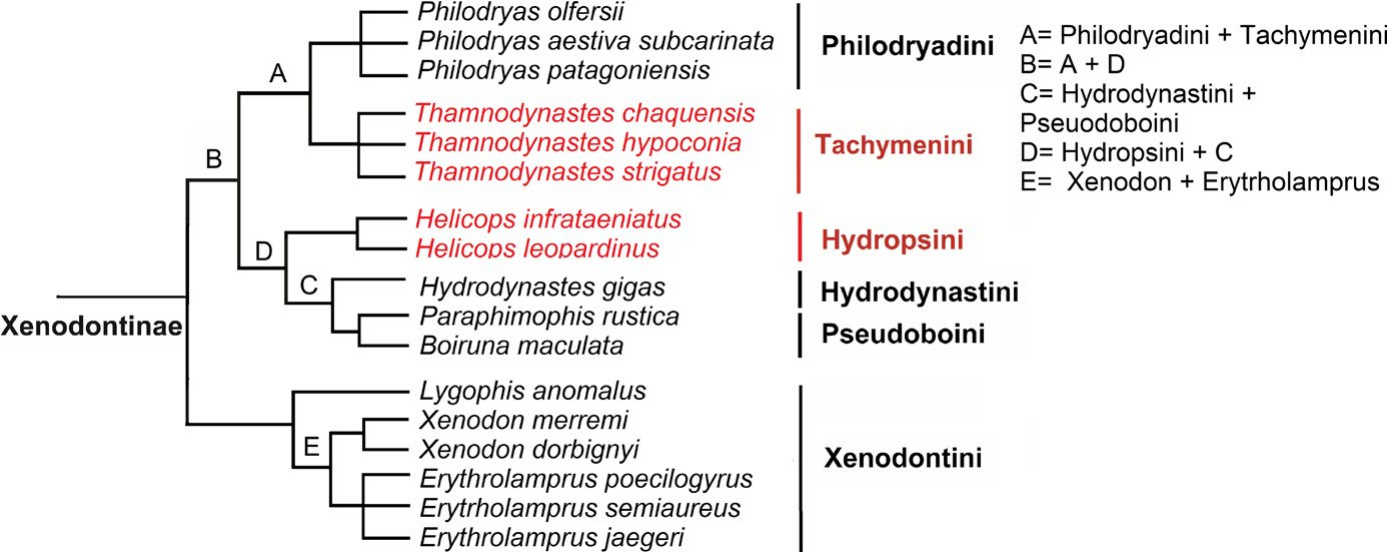
Phylogenetic relationships among 17 species of snakes used in the analysis. Oviparous clades are showed in black, and viviparous clades are showed in red

Then, we used a phylogenetic comparative method, canonical phylogenetic ordination (CPO; Giannini, 2003), to determine the reproductive variation explained by historical factors (phylogeny), and its covariation with other factors (habitat use, substrate use). CPO is a form of canonical ordination that uses the nested set of clades to which the taxa of the main matrix belong as an external matrix. In this application, CPO was a variance–covariance RDA, again with the reproduction matrix as the main matrix. The external matrix (phylogenetic matrix) consisted of a set of binary variables coding the clade membership of each individual and species (assigning 0s and 1s based on whether species belong to a group or not). The phylogenetic matrix was constructed using the phylogeny proposed by Grazziotin et al. (2012), as shown in Figure 1.

The significance of clade variables was first tested individually using 9999 unrestricted Monte Carlo permutations. A forward stepwise selection of clades from the tree matrix was then performed in order to obtain the reduced tree matrix that maximally explains the historical share of reproductive variation (see Giannini, 2003). Finally, using the same multivariate approach, we tested for the possible covariation of ecology and phylogeny using partial CPO (pCPO) (Giannini, 2003). In our example, the variation explained is partitioned into three components: ecology alone, clades alone, and their covariation. All ordinations were computed using Canoco for Windows 4.5 (ter Braak & Smilauer, 1998).

## 3 Results

When comparing the reproductive attributes between oviparous and viviparous snakes, some patterns were found. Low and medium reproductive potential did not seem to be related to the reproductive mode or frequency (Table 1). However, high reproductive potential seemed to follow a pattern, as it only occurred in oviparous species with annual reproductive frequency (Table 1).

Monte Carlo permutation tests on ecomorphological and phylogenetic matrices reduced the number of significant groups to be included in the pCPO model. The RDA using the ecological matrix resulted in only one classification, the aquatic variable, significantly explaining some fraction of the ecological variation (24.2% of total inertia; *F*_1_ = 6.1; *p = *.01). This variable was subsequently used in partial CPO analysis. The first two axes of this analysis explained 97% of the variation.

Monte Carlo permutations revealed a significant phylogenetic effect on the reproductive characteristics of the species studied. The CPO showed that phylogeny explained 67.4% of the variation in reproduction, whereas 32.6% of the variation remained unexplained. The first two axes of this analysis explained 73% of the variation. The clade with the greatest variability was the tribe Hydrodynastini (43.1%), to which the aquatic species *Hydrodynastes gigas* belongs. The following significant clade was the tribe Hydropsini (20.6%). The last clade contributing to significant reproductive divergence was the genus *Xenodon* (9.8%) (Table 2). These three significant clades were those used in the pCPO analysis.

**Table 2:** Results of canonical phylogenetic ordination for reproduction of 17 species in a temperate South American snake community

Taxa	Contribution (%)	*F*	*p*
Hydrodynastini	43.1	11.4	.05
Hydropsini	20.6	8	.01
*Xenodon*	9.8	4.8	.03

Clades are ranked by amount of variation explained at each node. Percentage of the contribution (relative to explains variation—67.4% –); and *F* and *p* values for each variable are given (9,999 permutations were used) for each main matrix. Note that no groups used for selection of variables yielded individual *p *≤ .05.

**Figure 2:**
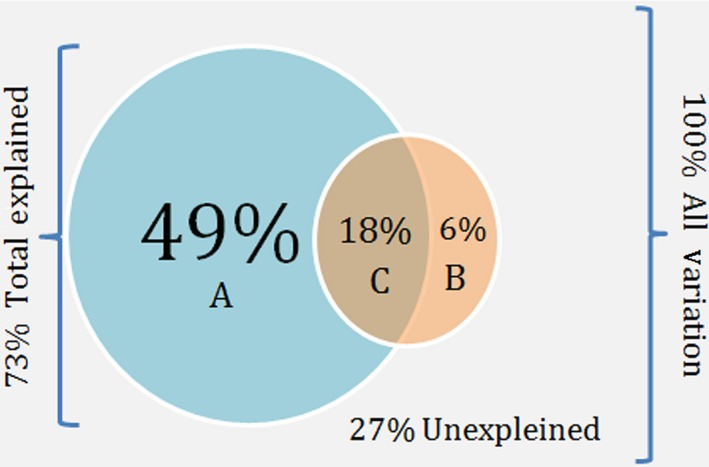
Diagram from a partial canonical phylogenetic ordination (pCPO). Compared groups are represented by circles, and the letters represent individual estimated fractions. A, exclusive variance of phylogeny; B, exclusive variance of ecology; C, shared variance

**Figure 3:**
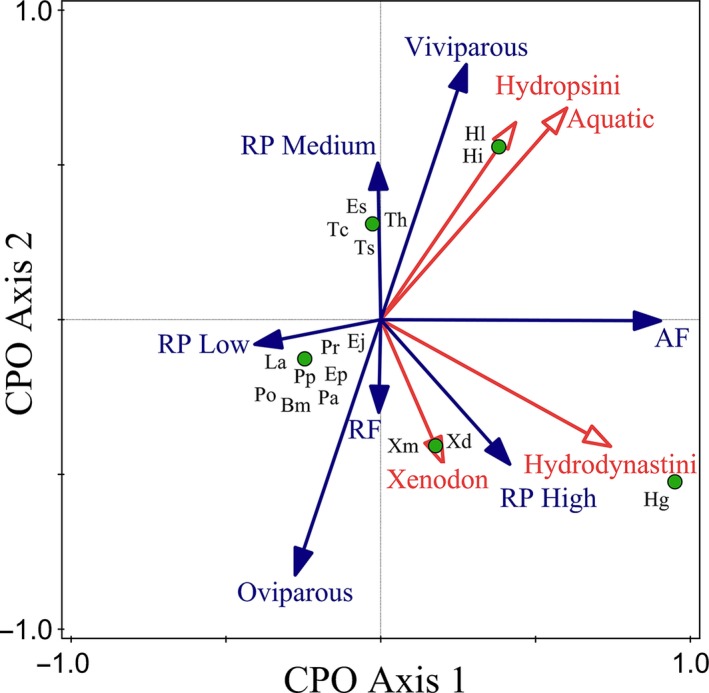
Triplot of snake reproduction from a partial canonical phylogenetic ordination (pCPO). Ecological and phylogenetic variable arrows (red): Each arrow points in the direction of the steepest increase of variable values. The angle between arrows indicates the correlation between individual variable. Reproduction variable arrows (blue): Each arrow points in the direction of the steepest increase of the values for corresponding reproduction variable. The angle between arrows indicates the sign of the correlation between the reproduction variables: The approximated correlation is positive when the angle is sharp. Species are represented by green circles. Bm, *Boiruna maculata;* Ej, *Erythrolamprus jaegeri;* Ep, *Erythrolamprus poecilogyrus;* Es, *Erythrolamprus semiaureus;* Hg, *Hydrodynastes gigas;* Hi, *Helicops infrataeniatus;* Hl, *Helicops leopardinus;* La, *Lygophis anomalus;* MF, mean fecundity; Pa, *Philodryas aestiva;* Po, *Philodryas olfersii;* Pp, *Philodryas patagoniensis;* Pr, *Paraphimophis rustica;* RP, reproductive potential; Tc, *Thamnodynastes chaquensis;* Th, *Thamnodynastes hypoconia;* Ts, *Thamnodynastes strigatus;* Xd, *Xenodon dorbingyi;* Xm, *Xenodon merremii*

The partial CPO showed that the overall variance in reproduction explained by ecology and phylogeny was 73% (*F*_4_ =  12; *p = *.0005); this variance was partitioned into variances unique to ecology (6%; *F*_1_ = 3.8; *p = *.06), unique to phylogeny (49%; *F*_3_ = 10.2; *p = *.001), and the shared variance (18%). The remaining variance was unexplained (27%) (Figure 2). A plot of this analysis showed that the CPO axis 1 divided the oviparous from the viviparous species, because these reproductive variables were negatively correlated (Figure 3). In addition, the plot revealed that viviparous species were positively correlated with medium reproductive potential and mean fecundity, while the oviparous ones were positively correlated with high and low reproductive potential. On the other hand, the aquatic ecological variable was highly and positively correlated with the tribe Hydropsini, but not as strongly, though positively correlated, with Hydrodynastini. On the contrary, the only significant ecological variable (aquatic) was negatively correlated with *Xenodon*, a genus composed of terrestrial and fossorial species (Table 1).

When the response variables (reproduction) were evaluated together with the explanatory variables (ecology and phylogeny), more comprehensive results were obtained. On the one hand, there was a high positive correlation between the tribe Hydropsini and the viviparous reproductive mode, whereas the correlation was positive, though not as strong with the mean fertility, and weaker but still positive with the medium reproductive potential. On the other hand, the correlation of the tribe Hydrodynastini was high and positive with the mean fecundity, as it also was with the oviparous reproductive mode. Finally, we obtained near‐zero correlation of both tribes with reproductive frequency, although it was positive for Hydrodynastini and negative for Hydropsini. The aquatic variable was highly and positively correlated with the viviparous reproductive mode, while the correlation was high but negative with the oviparous mode. Finally, we can see in the plot how the different species were associated with different reproductive, ecological, and phylogenetic variables (Figure 3).

## 4 Discussion

Retrieving the evolutionary history of Xenodontinae using ecological clues is a very difficult task, as they display such a high degree of plasticity that their history is almost “erased” whichever trait is considered (i.e., hemipenis, maxillary dentition, habitat) (Vidal et al., 2000). However, the characteristics of the species are determined by their history, and the events that occurred in the remote past may have strongly influenced much of the squamate biodiversity observed today (Bellini et al., 2015; Cadle & Greene, 1993; Colston et al., 2010; França et al., 2008; Vitt & Pianka, 2005; Vitt, Pianka, Cooper, & Schwenk, 2003). Hence, comparing ecological data of species in a phylogenetic framework, allow us to investigate whether there is a relationship between ecological and phylogenetic similarities. (Losos, 2008). The assemblage studied is, to a significant extent, the result of an admixture of evolutionarily clades, each contributing a set of species with different reproductive traits, giving the assemblage a particular and complex phylogenetic structure. Our results suggest that the reproductive characteristics strongly depend on the PH of each species, reflecting the clade to which it belongs. Our findings also provide new evidence of how the evolutionary history is embodied in the traits of living species, as other studies have already shown (Bellini et al., 2015; Cadle & Greene, 1993; França et al., 2008; Kraft, Cornwell, Webb, & Ackerly, 2007; Webb, Ackerly, Mcpeek, & Donoghue, 2002). However, the EF also influenced the reproduction of South American Xenodontines, although to a lesser extent. According to Shine (2003), reproductive tactics are clearly linked to features of the environment or of the species’ morphology and ecology.

The prevalent reproductive mode of the Xenodontinae subfamily is mostly oviparous, with few viviparous species (see Pizzatto et al., 2008a). The reproductive mode determines other reproductive characteristics of a snake's life history (Shine, 2003). When comparing the reproductive frequency, an important difference arose between oviparous and viviparous species. Oviparous species had an annual frequency of reproduction; that is., the same individual could potentially reproduce every year (Giraudo et al., 2007, 2014). By contrast, the viviparous had longer reproductive cycles, it being practically impossible for a single female to reproduce in two consecutive years (Bellini et al., 2013, 2014; Gregory, 2009; Ibarguengoytia & Casalins, 2007; Shine, 2003). The biennial reproductive frequency is probably a consequence of late parturition (Edwards, Jones, & Wapstra, 2002; Ibarguengoytia & Casalins, 2007).

The reproductive season of most reptiles of the Middle Paraná River starts at the beginning of spring and extends throughout the summer (Giraudo et al., 2007). Viviparous species give birth to their young between summer and early autumn, and the available time to accumulate energy before winter is very short (Bellini et al., 2013, 2014). On the other hand, the oviparous species of our assemblage oviposit their eggs in spring, so the time to accumulate energy for the next breeding season is much longer, enabling annual egg laying (Giraudo et al., 2007, 2014).

In addition, viviparity is considered a strategy for species that live in aquatic and unpredictable habitats (Giraudo et al., 2007; Shine, 1985). It is not surprising that the two viviparous tribes of our community are composed of aquatic species, or that they adjust their reproductive cycle to the hydrological cycles of the Parana River (Bellini et al., 2013, 2014; Giraudo et al., 2007). Most births and neonates of aquatic species (Tachymenini and Hydropsini) are found in March (early autumn) before spring or summer, coinciding with the maximum historical values in the hydrometric and precipitation cycles (see previous works: Giraudo et al., 2007; Bellini et al., 2013, 2014). It is probable that newborns and gravid females of these aquatic snakes could find a greater availability of aquatic environments in floodplain lakes and marshes, optimizing the possibilities to find refuge and feeding areas (Giraudo et al., 2007). This was evident in the graph, in which a high association of reproductive mode with the aquatic variable (positive with the viviparity and negative with the oviparity) was observed. This may be because, within South American Xenodontines, all nonterrestrial macrohabitat associations have evolved repeatedly (Cadle & Greene, 1993). Additionally, it appeared that each different tribe of South American Xenodontines was able to invade many ecological niches (Vidal et al., 2000). For example, the sister group to Pseudoboini, the genus *Hydrodynastes*, is aquatic while members of the Pseudoboini tribe are mainly terrestrial and arboreal (Gaiarsa, de Alencar, & Martins, 2013; Giraudo et al., 2014).

Despite the influences of EF on reproduction, phylogenetically related snakes were found to have more similar reproductive traits among them than with those species that are not related. When performing the analysis, these concepts became even more evident. We found significant reproductive differences among the clades that compose our assemblage due mainly to phylogenetic causes. The effect of PH was always greater than the effect of EF. In the case in which ecology and phylogeny were evaluated separately, the former explained only 24% of the variation, whereas the second explained 67% of the variation. On the other hand, it is not accidental which variables were significant in both analyses. In the CCA, the only significant ecological variable was the aquatic use of the substrate, while the first two variables that contributed the most (63%) from the phylogeny were two tribes (Hydrodynastini and Hydropsini) composed of aquatic species. Besides, it is also noteworthy that one of the tribes was composed of an oviparous species (Hydrodynastini) and the other, several viviparous forms (Hydropsini). Further elucidation of the effects of phylogeny was seen in the pCPO analysis, which accounted for 49% of the variability of reproductive characteristics in the assemblage, whereas the ecology only explained 6%. Nevertheless, together they explained 18% of the variability; which means that some of the variability in reproduction was determined by both the phylogeny and ecology. However, 27% of the variability in reproductive characteristics still could not be explained by PH, EF or a combination of both.

Evolutionary history is believed to largely influence similarity across lineages (Cavalheri, Both, & Martins, 2015; França et al., 2008; Losos, 2008), and so was demonstrated by our results. We found that the reproductive traits under study had a strong phylogenetic signal in South American xenodontines of a temperate region of South America, the ancestry playing a relevant role in reproduction. The EF also influenced the reproduction, although to a lesser extent. Furthermore, this same pattern is repeated in other important biological attributes such as diet, at least in part of the same community (Bellini et al., 2015). This allows us to suggest that this is a generality that may be applied to other life‐history traits. Finally, although the EF provided some explanation, we can say that evolutionary history, more than ecology, appears to have played a profound role in determining the reproduction of our temperate snake community. It is expected that the same pattern occurs in others clades of snakes, but it should be tested in different communities of snakes from around the world. Our findings provide new evidence of how the evolutionary history is embedded in the traits of living species, even at the end of the world.

## Conflict of interest

None declared.
